# Neuromodulation of Cortical Targets in Freezing of Gait

**DOI:** 10.1111/ejn.70406

**Published:** 2026-01-23

**Authors:** Gonzalo J. Revuelta, Daniel Lench, Carla Silva‐Batista, Marian L. Dale, Martina Mancini

**Affiliations:** ^1^ Department of Neurology Medical University of South Carolina Charleston South Carolina USA; ^2^ Oregon Health Sciences University Portland Oregon USA; ^3^ Portland VA Medical Center PADRECC Portland Oregon USA

**Keywords:** cortical control, freezing of gait, neuromodulation, Parkinson's disease, volitional control

## Abstract

Freezing of gait (FOG) is a disabling feature of Parkinson's Disease (PD) with unclear underlying pathophysiology. Evidence from multimodal neuroimaging studies suggests that complex interactions between cortical and subcortical areas may occur in FOG. While noninvasive neuromodulation techniques, such as transcranial magnetic stimulation (TMS), can effectively modulate large‐scale networks involved in FOG, the development of noninvasive neuromodulation interventions is limited by an incomplete understanding of the interactions between underlying network disruptions and FOG behavior. Recent studies have brought into question whether observed network changes in FOG are truly causal or secondary, and if secondary, are they adaptive, maladaptive, or not related? Although these questions go beyond correlative analyses, neuromodulation approaches provide an opportunity to systematically alter networks involved in FOG, providing evidence of a causal relationship. Here, we present evidence from noninvasive neuromodulation interventions of multiple cortical targets and their effects on behavior. In an attempt to leverage prior work to shed light onto the pathophysiology of FOG, we provide specific definitions of key aspects of gait behavior. We also aim to provide a framework under which adaptive and maladaptive network changes can be interpreted and targeted for the development of neuromodulation interventions. We encourage the design of future neuromodulation studies to consider including multimodal outcomes that will expand our understanding of the relationship between FOG behavior and treatment related network changes.

## Graphical abstract

Above is a graphical representation of a systematic approach to evaluate the role of cortical regions in the development of FOG using neuromodulation. Systematic neuromodulation of cortical targets can inform whether these network changes are primary (causal) or secondary. (A) In this example rTMS is focused on the engagement of cortical brain regions (red) responsible for volitional control of gait (SMA; Supplemental Motor Area, CMA; Cingulate Motor Area, M1; Primary Motor Cortex). These regions have connectivity with locomotor centers important for gait automaticity (SLR; Subthalamic Locomotor Region, CLR; Cerebellar Locomotor Region, and PPN: Pedunculopontine Nucleus). This ultimately impacts downstream central pattern generators (CPGs) in the spinal cord which regulate gait cycles. (B) In this example, specific rTMS parameters (1 Hz, 10 Hz, cTBS, and iTBS) can be manipulated to have distinct effects on cortical circuits allowing us to infer the adaptive or maladaptive contributions of these targets. (C) In cases where there is no clear impact of neuromodulation of FOG behavior, other targets can be evaluated and alternative models of how cortex contributes to FOG can be considered. For example the crosstalk model proposes disrupted neural signals from limbic and cognitive regions in addition to motor circuits.

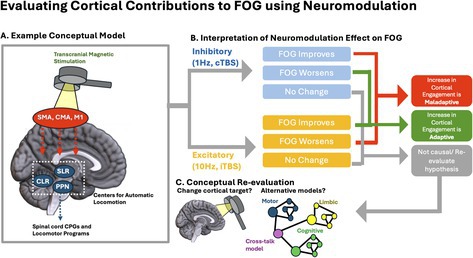

**Abbreviations**
APAs: anticipatory postural adjustments
cTBS: continuous theta burst stimulation
DLPFC: dorsolateral prefrontal cortex
FAB: Frontal Assessment Battery
FOG: freezing of gait
FOGQ: Freezing of Gait Questionnaire
iTBS: intermittent theta burst stimulation
M1‐LL: primary motor cortex corresponding to the lower limb
MMSE: mini mental status examination
mPFC: medial prefrontal cortex
PD: Parkinson's disease
rTMS: repetitive transcranial magnetic stimulation
RCT: randomized controlled trial
rs‐fMRI: resting state functional magnetic resonance
SMA: supplementary motor area
SMC: supplementary motor cortex
tDCS: transcranial direct current stimulation
TUG: Timed Up and Go
UPDRS: Unified Parkinson's Disease Rating Scale

## 1 Background

Under the prevailing current pathophysiological model of Parkinson's disease (PD), degeneration of dopaminergic neurons in the substantia nigra pars compacta produces marked dopaminergic denervation of the posterior putamen. This region is critical for habitual motor control; thus, its dysfunction results in impaired movement automaticity and increased reliance on goal‐directed, attention‐demanding motor control (Redgrave et al. 2010). Imaging studies support this model: healthy individuals consistently recruit the posterior putamen during automatic movements, whereas individuals with PD show a shift toward activation of more rostral striatal regions and frontoparietal cortical regions when performing the same tasks. As a result, individuals with PD rely on executive resources to regulate gait, a process normally executed automatically (Wu et al. 2015).

Within this framework, freezing of gait (FOG) can be viewed as a loss of automatic control of locomotion that must be compensated by volitional control until the system becomes overwhelmed, for example, during dual‐tasking, high cognitive load, or heightened limbic drive (Lewis and Shine 2016; Georgiades et al. 2019; Shine, Matar, Ward, Bolitho, et al. 2013). Multiple conceptual models have been proposed to explain this breakdown (Nutt et al. 2011), including the *interference model*, in which competing cognitive–motor demands overload limited executive control capacity (exemplified above), and the *reduced capacity model*, which posits deficits across multiple domains/networks (Lewis and Shine 2016; Georgiades et al. 2019; Shine, Matar, Ward, Bolitho, et al. 2013). Here, we focus on how neuromodulation of cortical targets can be used to address fundamental questions about the FOG pathophysiology and to guide future therapeutic development.

In order to interpret the network changes that have been reported in FOG (e.g., altered frontoparietal activation, disrupted cortico‐striatal and cortico‐brainstem coupling) it is critical to understand if such changes are **compensatory adaptations** (either effective or ineffective) or truly **maladaptive** responses that contribute to FOG severity. Furthermore, behavioral associations to imaging findings remain unclear, as it is unknown whether the observed imaging findings are primary (causal) drivers of FOG or secondary consequences of gait breakdown. To address these knowledge gaps, neuromodulation strategies have been utilized to either strengthen or weaken cortical–subcortical functional connections, network synchrony, coherence, or coupling, testing varied hypotheses and conceptual models. It is important to recognize that the interactions that occur within these networks are complex; however, evidence so far suggests the presence of systems‐level dysfunction in FOG and neuromodulation interventions currently available can effect network changes leading to measurable behavioral effects.

## 2 Definitions

### 2.1 Gait Automaticity

The ability of the central nervous system to control walking with minimal use of executive control (which demands attention) (Clark 2015). Proxies of loss of gait automaticity in PD include increased prefrontal cortex (PFC) activity during walking, higher dual‐task cost of gait, and greater gait variability. In fact, people with FOG usually show higher PFC activity (Vitorio et al. 2020; Bardakan et al. 2022), higher dual‐task cost of walking (de Souza Fortaleza et al. 2017; Monaghan et al. 2023), and greater gait variability (Vitorio et al. 2020; Hausdorff, Balash, and Giladi 2003; Nanhoe‐Mahabier et al. 2011) compared to people without FOG.

### 2.2 Volitional Control

Motor tasks that are deliberate and attention dependent require significant executive resources.

### 2.3 Adaptive Network Responses

Any measurable network change that occurs as a result of FOG behavior can be considered adaptive. Adaptive changes are understood to be secondary, as opposed to primary or causal. Adaptive changes can be effective, which would imply that interventions to strengthen these changes can improve FOG behavior; ineffective, which would imply that interventions to increase or decrease these observed changes are unlikely to have any effect on FOG behavior.

### 2.4 Maladaptive Network Responses

Measurable network changes that occur as a result of FOG can also worsen FOG. Maladaptive network responses can be viewed as bad habits that need to be broken in order to facilitate rehabilitation or a positive behavioral change. Neuromodulation interventions targeting maladaptive changes would aim to reverse these changes in order to improve FOG behavior.

## 3 Electrophysiologcal and Imaging Evidence for Maladaptive Network Response in FOG

Highly automatized motor skills which have been learned over time (including locomotion) require little to no volitional control. In fact, volitional control may be disruptive in the context of such behaviors. For example, skilled athletes frequently experience performance deterioration when they “overthink” a well learned skill, an analogy relevant to PD and FOG. Conceptually, gait becomes increasingly attention dependent, requiring recruitment of cognitive resources that were not needed when automaticity was intact. Although this shift may initially represent a beneficial adaptation, excessive cognitive monitoring of gait may ultimately become maladaptive in a manner similar to overfocused motor control in skilled athletes.

At the circuit‐level, overreliance on cortical structures involved in volitional control of gait may interfere with brainstem and basal ganglia locomotor circuits that normally drive automatic gait. Anatomical studies demonstrate direct projections from the premotor and supplementary motor area (SMA) to subcortical locomotor centers, including components of the mesencephalic locomotor region (MLR) (Matsumura et al. 2000). Electrophysiological recordings in humans have shown that the pedunculopontine nucleus (PPN), a key structure within the MLR, interacts with the SMA as evidenced by changes in beta coherence prior to movement initiation (Tsang et al. 2010). While some degree of cortical‐brainstem coupling is likely necessary for adaptive modulation of gait, several studies report excessive coupling or abnormally increased SMA–PPN connectivity in individuals with FOG (Lench et al. 2020; Lench et al. 2021; Fling et al. 2014).

Several neuroimaging studies (fMRI) implicate frontal and frontostriatal dysfunction in FOG (Shine, Matar, Ward, Bolitho, et al. 2013; Gilat et al. 2017; Shine, Matar, Ward, Frank, et al. 2013; Ehgoetz Martens et al. 2018). FOG appears to arise when the balance between automatic motor control and cognitively mediated control processes destabilizes (Vandenbossche et al. 2012) through impaired integration of cognitive control networks (e.g., executive and attentional networks) (Shine, Matar, Ward, Frank, et al. 2013; Shine, Moustafa, Matar, Frank, and Lewis 2013). Virtual reality fMRI gait paradigm consistently shows heightened frontal and parietal activation combined with reduced activity in subcortical regions during FOG episodes (Shine, Matar, Ward, Bolitho, et al. 2013; Gilat et al. 2017; Shine, Matar, Ward, Frank, et al. 2013). In addition, FOG episodes (off medication) are associated with reduced synchrony across corticostriatal pathways (Ehgoetz Martens et al. 2018).

Increasingly complex models have considered how multi‐domain networks can contribute to FOG behavior. The “cross‐talk” model of FOG, for example, suggests that excessive communication and conflict between cognitive, motor, and limbic networks can lead to FOG. Neural networks, which are usually segregated in healthy controls, can become over‐integrated during pathological “freezing” states. This integration appears to increase during times in which PD patients are in their OFF medication state (Shine et al. 2019). However, neurotransmitters other than dopamine are also likely involved in changes in pathological network integration. For example, the noradrenergic system is thought to drive the integration of networks by increasing neural gain across the entire brain. Furthermore, these topological features of networks are dynamic and fluctuate over time, which aligns with the episodic and paroxysmal nature of FOG (Shine et al. 2016). Overall, this conceptual framework provides a plausible explanation of how high levels of anxiety, arousal, and cognitive load may influence cortical networks to disrupt the effectiveness of automatic gait circuits to function properly (Taylor et al. 2022). At this time, causal studies are needed to determine the precise extent to which network integration is maladaptive or adaptive. Furthermore, it remains unknown what levels of network integration or segregation are optimal for gait performance.

A major limitation of fMRI studies in investigating cortical contributions to gait is that subjects cannot ambulate in the scanner environment. Mobile neuroimaging methods, such as functional near infrared spectroscopy (fNIRS), have been used to study cortical activation, more commonly PFC activation, during walking and turning in people with PD with and without FOG (Vitorio et al. 2020; Ranchet et al. 2020; Hamacher et al. 2015; Herold et al. 2017; Maidan et al. 2016; Silva‐Batista et al. 2024; Belluscio et al. 2019). Gait during dual‐task conditions and turning in place have been the main triggers for evoking FOG in the laboratory setting (Conde et al. 2023). Using fNIRS, several reports show increased PFC activity during dual‐task walking (Vitorio et al. 2020), 360° turning in place (Belluscio et al. 2019), and immediately before and during FOG episodes triggered by an unanticipated 180° turning while walking (Maidan et al. 2015). Recent work expanding beyond the PFC demonstrated that freezers also show elevated activation in SMA, premotor cortex, and posterior parietal cortex during gait‐related tasks (turning, stopping while walking). However, during actual FOG episodes, cortical activation shows a distinct and abnormal pattern compared to voluntary stopping: PFC activity decreases markedly while SMA, premotor cortex, and posterior parietal cortex do not show a significant change, suggesting that no cortical region effectively “steps in” during the freeze. This pattern suggests that FOG may be driven by a disruption or imbalance within the stopping‐related network, particularly between SMA and PFC, leading to a failure of coordinated cortical–subcortical control at the moment of freezing.

Taken together, these fMRI and fNIRS findings suggest that people with FOG demonstrated increased activity in fronto‐parietal regions in an attempt to compensate for striatal dysfunction leading to reduced automaticity of gait and increased stride‐to‐stride variability (Hausdorff, Schaafsma, et al. 2003; Gilat et al. 2013; Plotnik et al. 2005). Reliance on these cognitive control networks aligns with evidence that people with PD increasingly depend on executive resources and external cues in order to generate effective locomotion by switching to goal‐directed behavior (Redgrave et al. 2010; Gilat et al. 2021). However, this compensatory strategy may become inefficient in two cases: (1) during challenging conditions that further rely on PFC, such as when multi‐tasking or during turns, when the load on the frontal networks may become unsustainable (overload), thus leading to FOG (Shine, Matar, Ward, Frank, et al. 2013; Vandenbossche et al. 2012; Shine, Moustafa, Matar, Frank, and Lewis 2013) or (2) the degeneration of the compensatory cortical areas that occurs with disease progression. A recent review suggests that compensatory recruitment of cognitive and sensory networks initially supports gait, but deteriorates over time, leading to more frequent breakdown of gait and FOG (Tosserams et al. 2025). While studies show correlations between worsening FOG and greater cortical control over gait (Belluscio et al. 2019; Maidan et al. 2015; Shine et al. 2014), it remains unclear if this is due to worsening FOG, which engages greater cortical areas, or truly a maladaptive process that contributes to worsening FOG severity. To understand this relationship, *manipulating cortical targets systematically* via *neuromodulation approaches can be highly informative*.

## 4 Review of Neuromodulation Interventions Designed to Improve Adaptive Responses in FOG

A comparative analysis of the available evidence on neuromodulation of cortical control is challenging, given the high number of variables, including neuromodulation modality, study design, target, and choice of FOG outcome measures. We sought to represent neuromodulation modalities that addressed FOG specifically in idiopathic PD and excluded open‐label studies as it is difficult to draw conclusions from these findings. The objective of this summary was not to evaluate clinical evidence toward efficacy, but rather to elucidate the effects of experimental manipulation of relevant networks on FOG severity.

Overall, improvements reported from excitatory repetitive transcranial magnetic stimulation (rTMS) or transcranial direct current stimulation (tDCS) approaches were modest, with up to 2 points improvement in the Freezing of Gait Questionnaire (FOGQ), reduction of two FOG episodes, 4% reduction of percent time frozen in FOG provoking tasks, and one study showed up to a 15‐s reduction in TUG time in the active group but no significant improvement in FOGQ. Several excitatory studies did not show any efficacy or were equivocal (see Table 1).

**TABLE 1:** Randomized controlled trials of noninvasive neuromodulation interventions are tabulated and compared. Studies are categorized according to the type of intervention modality and efficacy. Each modality is then sub‐categorized based on whether or not the intervention is excitatory or inhibitory.

Excitatory studies that improve FOG									
TMS									
	Modality	Design	*N*	Number of sessions	Dose (total pulses/current)	Target	Outcome measures	Significant results	Durability
Kim et al., 2015 (Kim et al. 2015)	10 Hz rTMS vs. Sham	RCT	17 PD with FOG	5 sessions	5000 pulses	Primary Motor Cortex (M1) leg area	180 Turns FOGQ TUG UPDRS	1.3‐step improvement in the 180° turn test. 1.6‐point improvement in FOG‐Q. 2‐s improvement in TUG. 1.4‐point improvement in UPDRS‐III.	All significant improvements were maintained 1 week after intervention.
Mi et al. 2019 (Mi et al. 2019)	10 Hz rTMS vs. Sham	RCT	30 PD with FOG	10 sessions	10,000 pulses	SMA	FOGQ UPDRS Turn duration	Immediate 1.6‐point improvement in FOG‐Q after intervention 2.13‐point improvement in FOG‐Q at 4 weeks post‐intervention	Improvements in FOGQ persisted at least 4 weeks after intervention.
Potvin‐Desrochers et al. 2023 (Potvin‐Desrochers et al. 2023)	iTBS, cTBS, Sham	RCT	14 PD with FOG	4 sessions	Not Specified	PPC	Noninstrumented FOG‐provoking task	iTBS: Decrease in the number of freezing episodes from a median of 6 to 4 episodes Reduction in percent time frozen during the task from 14% to 10% cTBS and Sham: No improvements	Not assessed.
Lee et al. 2014 (Lee et al. 2014)	10 Hz rTMS vs. Sham	RCT, Crossover	20 PD with FOG	Single session	1000 pulses	SMA M1‐LL DLPFC On dominant hemisphere	TUG Turn time and Step number UPDRS FOGQ	TUG: M1‐LL stimulation reduced TUG time from 52.7 ± 13.0 s to 38.0 ± 5.8 s. DLPFC stimulation reduced TUG time from 47.3 ± 9.7 s to 42.0 ± 8.6 s No significant changes in FOGQ score	Not assessed
tDCS									
Dagan et al. 2018 (Dagan et al. 2018)	Anodal tDCS vs. Sham	RCT, Crossover	20 PD with FOG	3 sessions (20 min each): M1 only M1 + DLPFC Sham	2 mA continuously over 20 min	M1 Left DLPFC	FOG provoking task TUG Stroop test	Significant reduction in FOG episodes during simultaneous M1 + Left DLPFC tDCS compared to M1 only or sham, 88% of participants (15 out of 17) showed improvement in FOG. Exact amount of reduction not specified	Not assessed.
Valentino et al. 2014 (Valentino et al. 2014)	Anodal tDCS vs. Sham	RCT, Crossover	10 with FOG	5 consecutive daily sessions	2 mA continuously over 20 min	M1	Stand Walk Sit (SWS) FOGQ Gait & Fall Questionnaire UPDRS	SWS: Significant reduction in the number and duration of FOG episodes. Reduction in the number of steps and time needed to complete the SWS. FOGQ, UPDRS, Gait & Fall Questionnaire: Significant improvement	1 month
Excitatory Studies that do not improve FOG									
TMS									
Brugger et al. 2020 (Brugger et al. 2020)	iTBS vs. Sham	RCT	23 (12 PD with FOG, and 11 PD without FOG)	Not Specified	Not Specified	BL SMC	3D gait analysis (stride time, stride length) FOGQ UPDRS FAB MMSE	Deterioration: iTBS over the SMC resulted in a relative worsening of gait initiation parameters in PD patients with FOG. Numerical values were not reported	Not assessed.
Tard et al. 2016 (Tard et al. 2016)	iTBS vs. Sham	RCT, Crossover	15 PD with FOG	2 sessions (one iTBS, one sham)	600 pulses over 3 min		Percent time with FOG 3D analysis	No improvement	Not assessed.
Rektorova et al. 2007 (Rektorova et al. 2007)	10 Hz rTMS vs. Sham	RCT	6 PD with FOG	5 Consecutive daily sessions	6750 pulses	M1 L DLPFC	Video analysis UPDRS Neuropsychological Battery	No improvement	Terminated due to patient withdrawal.
tDCS									
Lu et al. 2018 (Lu et al. 2018)	Anodal tDCS vs. Sham	RCT, Crossover	10 PD with FOG	2 sessions (one tdcs one sham)	1 mA continuously for 10 min	SMA	APAs	No improvement	Not assessed.
Combined TMS/tDCS									
Chang et al. 2017 (Chang et al. 2017)	10 Hz rTMS and Anodal tDCS vs. Sham	RCT	32 PD patients (16 dual‐mode stimulation, 16 rTMS only)	5 sessions	rTMS: 5000 pulses total tDCS: 1 mA for 20 min	rTMS: M1‐LL tDCS: Left DLPFC	FOGQ 180 Turn UPDRS TUG	No improvements	Limited to 1 week after intervention.
Excitatory studies that are equivocal									
TMS									
Degan et al. 2017 (Dagan et al. 2017)	10 Hz rTMS vs. Sham	RCT	9 PD with FOG	16 sessions	33,600 pulses	mPFC	FOG provoking task NFOGQ UPDRS Spatiotemporals (gait speed, stride length, step time, swing time)	FOG Provoking task: Significant reduction in FOG severity Large effect sizes reported (0.90 after intensive phase, 0.82 after maintenance phase)	Not assessed.
tDCS									
Lee et al. 2021 (Lee and Kim 2021)	Anodal tDCS + Visual Cueing vs. Sham	RCT	30 PD patients (FOG was not a requirement; exact number with FOG not provided)	20 sessions	2 mA continuously for 20 min	SMA	FOGQ Functional Gait Assessment (FGA) Cadence UPDRS Spatiotemporal gait parameters (measured using GAITRite system)	FOGQ: No improvements Improvement in gait and cadence	Limited to 2 weeks after intervention ended
Manor et al. 2021 (Manor et al. 2021)	tDCS vs. Sham	RCT	77 PD with FOG	15 sessions	Not specified	Anodal tDCS: Left DLPFC Cathodal tDCS: BL M1‐LL	FOG Provoking task UPDRS nFOGQ	No overall improvement in FOG symptoms Subset of participants with mild FOG severity showed improvement	Increased daily living step counts observed after 2 weeks of stimulation and at 10‐week follow‐up
Inhibitory Studies that improve FOG									
Lench et al. 2021 (Lench et al. 2021)	1 Hz rTMS + Gait Training vs. Sham + Gait Training	RCT	20 PD with FOG	10 sessions	12,000	SMA	nFOGQ Spatiotemporals rs‐fMRI UPDRS	No statistically significant improvement in nFOG‐Q scores between active and sham groups Active rTMS reduced SMA connectivity to regions involved in executive control and attention	3 months

## 5 Review of Neuromodulation Interventions Designed to Diminish Maladaptive Response in FOG

While the vast majority of neuromodulation studies for FOG have been performed to increase cortical control, only two studies used rTMS to reduce cortical control of gait (Lench et al. 2021). One comparative study of cTBS and iTBS reported a small improvement in the iTBS (excitatory) group (2 freezing episodes or 4% reduction) and no improvement in the inhibitory group (Potvin‐Desrochers et al. 2023). The other study (Lench et al. 2021) (conducted by authors DL and GJR) tested the framework that hyper‐connectivity of cortical brain regions to automatic gait centers could be dampened by using an inhibitory form of stimulation. In this study, 20 participants with FOG were randomized into either a sham or active TMS treatment over 10 days. 1 Hz rTMS was administered at a suprathreshold level (110% rMT) over the SMA and combined with daily gait training sessions. These 20‐min training sessions were used to promote gait automaticity by having participants focus on a cognitive task while maintaining a constant gait velocity. This study found a 4.8‐point reduction on the New Freezing of Gait Questionnaire FOG‐Q (nFOG‐Q) following the active TMS intervention and a modest 1.8‐point reduction following sham TMS intervention which included gait training. Although pre‐ to post‐changes in the active group were significant, between‐group differences were not significant in this small sample. Reductions in SMA connectivity were found with several brain regions, including the anterior cingulate cortex (ACC), medial PFC, and angular gyrus in the active group. This study provides direct evidence that a reduction in cortical control of gait can lead to an improvement in FOG.

Taken together, there is reasonable evidence that reducing cortical control of gait through noninvasive forms of stimulation may in fact alleviate FOG. Future studies that compare stimulation protocols and perform more standardized interventions to facilitate gait automaticity will be needed to provide more conclusive evidence.

## 6 Conclusion

Here we explore the role of cortical (volitional) control of locomotion and present a conceptual framework in which modulating cortical input into subcortical locomotor centers may improve FOG. Given the development of noninvasive neuromodulation for cortical targets, we propose that neuromodulation interventions for FOG can address two important knowledge gaps: (1) is cortical activation, involving PFC, premotor, and SMA, an ineffective adaptation or a maladaptive response? and (2) should these changes be enhanced or inhibited to improve FOG? Currently, the vast majority of the literature in this field has aimed to increase cortical control of locomotion via neuromodulation and rehabilitation approaches with largely equivocal, negative, or subclinical effects. We propose that under this framework, cortical inhibitory strategies, either in isolation or combined with approaches to improve gait automaticity, deserve systematic investigation.

Although it is difficult to compare efficacy across studies due to differences in modalities, targets, design, and particularly the lack of standardized outcome measures, the FOGQ and nFOGQ have been most widely used, allowing for a more direct comparison of the magnitude of effects of multiple studies. The nFOGQ, however, has been criticized due to having a large minimum detectable change (Hulzinga et al. 2020), which is greater than any of the reported differences. It is important to note that the reported improvements in the limited number of inhibitory studies are at least as large as or greater in magnitude than the excitatory studies; however, future studies should include objective measures of FOG severity.

Interpreting neuromodulation effects is further complicated by the fact that inhibitory or excitatory strategies do not always result in the intended effect, particularly with more advanced or accelerated approaches like theta burst stimulation (TBS) (McCalley et al. 2021). As our understanding of the individual variability of FOG grows, and the variability of the effects of noninvasive neuromodulation interventions, future studies may pursue personalized interventions based on individual‐level findings that guide network‐specific interventions.

It is important to highlight that only one study (Hausdorff, Balash, and Giladi 2003) investigated the mechanistic effects of the intervention. This allows for accurate interpretation of the behavioral effects of the intervention. Clinical improvement (or lack thereof) of an investigational therapy can be a result of a variety of factors including: target, dose, modality, selected outcomes and directionality of the intervention. Therefore a null effect is not informative unless the study is designed to detect target engagement and the network effect of the intervention. If the network effect was normalized and there was no behavioral effect, this finding is much more meaningful as it provides critical data to clarify the relationship between the network change and FOG behavior itself. Thus far, there is evidence from one study that the SMA target can be engaged, and SMA connections can be decreased with rTMS, resulting in a positive effect on FOG behavior. There is also evidence of positive and null effects with excitatory neuromodulation interventions on a variety of targets, which are difficult to interpret with limited data on target engagement and network effects.

While current neuromodulation studies have primarily focused on either enhancing or inhibiting cortical control, we acknowledge that these do not encompass other more complex models that underlie FOG, such as the “cross‐talk” model. Despite this gap in research, we believe that modern neuromodulation techniques may also have the potential to interrogate the impact of cortical network features that have not been traditionally evaluated in a causal manner, such as network integration. To date, most cortical neuromodulation approaches for FOG have been focused on rTMS and tDCS. However, other neuromodulation approaches, like transcranial alternating current stimulation (tACS), which can entrain neural oscillations across the brain, have the potential to modulate the synchrony of neural activity across spatially distributed networks (Bachinger et al. 2017). Additionally, transauricular vagus nerve stimulation (taVNS) can transiently modulate noradrenergic brain centers such as the locus ceruleus, which have previously been shown to globally drive network integration and segregation (Wienke et al. 2023). These noninvasive neuromodulation approaches, paired with the advancement of more dynamic neuroimaging techniques, are a promising avenue to elucidate the adaptive or maladaptive role of cortical network integration and “cross‐talk” in FOG.

As the field advances toward accelerated stimulation paradigms, individualized targeting, and combined neuromodulation‐rehabilitation approaches, it will be critical to evaluate both behavioral outcomes of FOG and the underlying network effects. We encourage future studies to include neuroimaging and neurophysiological outcomes along with standardized behavioral outcomes for FOG. Such mechanistic–behavioral coupling will be essential for determining which cortical processes should be suppressed, enhanced, or rebalanced to meaningfully improve FOG.

## Author Contributions

**Gonzalo J. Revuelta:** conceptualization, data curation, formal analysis, funding acquisition, methodology, writing – original draft, writing – review and editing. **Daniel Lench:** conceptualization, writing – review and editing. **Carla Silva‐Batista:** writing – review and editing. **Marian L. Dale:** conceptualization, writing – review and editing. **Martina Mancini:** conceptualization, writing – review and editing.

## Funding

This work was supported by the National Institute of Neurological Disorders and Stroke, (1R01NS131396, R00NS131447).

## Conflicts of Interest

The authors declare no conflicts of interest.

## Data Availability

As this is a spotlight, the data that support the findings of this study are available in the main document itself, referenced, and tabulated in Table 1.
